# Preliminary Pharmacogenomic-Based Predictive Models of Tamoxifen Response in Hormone-dependent Chilean Breast Cancer Patients

**DOI:** 10.3389/fphar.2021.661443

**Published:** 2021-11-25

**Authors:** Carla Miranda, Macarena Galleguillos, Roberto Torres, Karla Tardón, Dante D. Cáceres, Kuen Lee, María A. Redal, Nelson M. Varela, Luis A. Quiñones

**Affiliations:** ^1^ Laboratory of Chemical Carcinogenesis and Pharmacogenetics, Department of Basic-Clinical Oncology (DOBC), Faculty of Medicine, University of Chile, Santiago, Chile; ^2^ National Cancer Institute, Santiago, Chile; ^3^ Institute of Population Health, Faculty of Medicine, University of Chile, Santiago, Chile; ^4^ Faculty of Medicine, University of Chile, Santiago, Chile; ^5^ Genetic Division, Department of Medicine, Hospital de Clínicas José de San Martín, Buenos Aires, Argentina; ^6^ Latin American Network for Implementation and Validation of Clinical Pharmacogenomics Guidelines (RELIVAF-CYTED), Madrid, Spain

**Keywords:** breast cancer, pharmacokinetics, pharmacodynamics, polymorphism, ADR, Relapse

## Abstract

Tamoxifen (TAM), a selective oestrogen receptor modulator, is one of the most used treatments in oestrogen receptor-positive (ER+) early and metastatic breast cancer (BC) patients. The response to TAM has a high degree of inter-individual variability. This is mainly due to genetic variants in *CYP2D6* gene, as well as other genes encoding proteins involved in the TAM pharmacokinetic and/or pharmacodynamic. Therefore, prediction of the TAM response using these genetic factors together with other non-genetic variables may be relevant to improve breast cancer treatment. Thus, in this work, we used genetic polymorphisms and clinical variables for TAM response modelling. One hundred sixty-two ER + BC patients with 2 years of TAM treatment were retrospectively recruited, and the genetic polymorphisms CYP2D6*4, CYP3A4*1B (CYP3A4*1.001), CYP3A5*3, UGT2B7*2, UGT2B15*2, SULT1A1*2, and *ESRA V364E* were analyzed by PCR-RFLP. Concomitantly, the therapeutic response was obtained from clinical records for association with genotypes using univariate and multivariate biostatistical models. Our results show that UGT2B15*1/*2 genotype protects against relapse (OR = 0.09; *p* = 0.02), CYP3A5*3/*3 genotype avoids endometrial hyperplasia (OR = 0.07; *p* = 0.01), SULT1A1*1/*2 genotype avoids vaginal bleeding (OR = 0.09; *p* = 0.03) and *ESRA 364E/364E* genotype increases the probability of vaginal bleeding (OR = 5.68; *p* = 0.02). Logistic regression models, including genomic and non-genomic variables, allowed us to obtain preliminary predictive models to explain relapse (*p* = 0.010), endometrial hyperplasia (*p* = 0.002) and vaginal bleeding (*p* = 0.014). Our results suggest that the response to TAM treatment in ER + BC patients might be associated with the presence of the studied genetic variants in *UGT2B15*, *CYP3A5, SULT1A1* and *ESRA* genes. After clinical validation protocols, these models might be used to help to predict a percentage of BC relapse and adverse reactions, improving the individual response to TAM-based treatment.

## 1 Introduction

Breast cancer (BC) is the second leading cause of cancer death in women (American Cancer Society, 2017) and is caused by many factors, such as age, breast density, heredity, and exposure to oestrogens (Kumar et al., 2010). Breast cancer (BC) patients with oestrogen receptor positivity (ER+) are treated in addition to surgery, chemotherapy, and/or radiation therapy with hormone therapy (Maughan et al., 2010; (McDonnell and Wardell 2010; NCI 2012). Tamoxifen (TAM), a type of hormonal therapy known as a selective oestrogen receptor modulator, behaves as an oestrogen antagonist in the breast and as an agonist in the endometrium (Lewis and Jordan 2005; Terán and Teppa 2005; Hertz et al., 2009; NCI 2012), and despite the new hormonal agents, TAM remains the drug of choice for any BC stage (Davies et al., 2011) since it increases the disease-free time in pre- or postmenopausal women, reducing the annual death rate by 34% (MINSAL, 2005; Untch and Thomssen, 2010). However, the response among patients is variable in terms of efficacy and side effects.

TAM undergoes extensive biotransformation in the liver by phase I (CYP) and phase II enzymes. Phase I generates, among other metabolites, N-desmethyltamoxifen (N-desmethylTAM), 4-hydroxy tamoxifen (4-hydroxyTAM), and 4-hydroxy-N-desmethyltamoxifen (endoxifen) (Brauch et al., 2009). Endoxifen and 4-hydroxyTAM are the main and unique active metabolites of TAM due to their high affinity to ERs and their activity being 30- to 100-fold greater than TAM itself (Wakeling and Slater 1980; Johnson et al., 2004; Fernández-Santander et al., 2007; Brauch et al., 2009). *In vitro* studies have determined that these metabolites are more effective in reducing cell proliferation (Jordan et al., 1977; Clarke et al., 2003; Borges et al., 2006). The two most abundant metabolites in plasma are N-desmethylTAM and endoxifen (Clarke et al., 2003). Subsequently, these metabolites undergo biotransformation by phase II enzymes, the main enzymes involved being SULT1A1, UGT2B7, and UGT2B15 (Nishiyama et al., 2002). TAM has a long half-life, and steady-state concentrations are obtained at 4 weeks for TAM and at 8 weeks for N-desmethylTAM (Buckley and Goa, 1989; Lien et al., 1992; Kisanga et al., 2004).

Differences have been found between the response to TAM and the histological type, degree of differentiation of the breast tumor cells, age, and state of menopause in the patient. TAM has an overall response rate of almost 75% in patients with ER+ and PR + BC. In the case of adjuvant treatment, TAM reduces the risk of relapse by 25% and mortality by 17% (Fisher et al., 1998; Davies et al., 2011). On the other hand, the long-term safety of TAM is well elucidated, and possible harmful effects on bone metabolism, endometrial cancer, thromboembolic diseases, and cognitive disorders due to chronic oestrogen deprivation in brain tissue have been described (Fisher et al., 1998; Cuzick et al., 2002; Cuzick, 2005). A 1% increase in the incidence of endometrial cancer has been observed due to its oestrogen-inducing effect in the uterus and thromboembolic events in 1–2% of cases.

Despite several studies having been conducted, after 3 decades, there are still differences in the TAM response that have not been explained. It is known that these variations in the response to drugs are multifactorial, the result of the interaction between multiple and complex genetic, physiological and environmental factors (Ma and Lu 2011). A potential explanation is the presence of genetic variants in the genes encoding biotransformation enzymes affecting their efficacy and safety (Zhou et al., 2008). In this respect, undoubtedly genetic variation in the *CYP2D6* driving to ultrarapid (XN), intermediate metabolizer or poor metabolizer phenotypes (e.g. *3, *4, *5, *9, *10, *17 haplotypes) may affects plasma concentrations of TAM and its metabolites, as CPIC has stablished in the updated guidelines (Goetz *et a*l, 2018). CYP2D6*4 (rs3892097) is associated with decreased side effects, such as hot flashes, disease-free time, and survival rate (Goetz et al., 2005; Jin et al., 2005; Schroth et al., 2007; Bijl et al., 2009; Ramón y Cajal et al., 2010; Rae et al., 2012), and decreased serum levels of TAM metabolites (Gjerde et al., 2007; Kiyotani et al., 2010; Lim et al., 2011; Madlensky et al., 2011). In addition, CYP3A4*1B (currently named CYP3A4*1.001, rs2740574) is associated with a 3-fold higher risk of endometrial cancer in TAM-treated BC patients (Chu et al., 2007). CYP3A5*3 (rs776746) is associated with characteristics of the tumor in BC postmenopausal women treated with TAM (Tucker et al., 2005). On the other hand, *CYP2C9* *2 and *3 variants reduce the concentration of TAM metabolites, affecting the therapeutic response (Mürdter et al., 2011).

Phase II enzymes also affect TAM response. SULT1A1*2 (rs9282861) increases the risk of relapse in TAM-treated BC patients (Wegman et al., 2005). UGT2B15*2 (rs1902023) decreases the risk of relapse of BC (Nowell et al., 2005). Furthermore, the presence of UGT2B15*2 and SULT1A1*2 is associated with a lower risk of relapse and a significant reduction in survival time in patients with BC treated with TAM (Nowell et al., 2005).

Finally, several mutations have been identified in the oestrogen receptor α gene (*ESRA*) in BC patients (Herynk et al., 2004); however, their effect on the efficacy and safety of TAM treatment has not been elucidated. In this respect, using SIFT and PolyPhen bioinformatic tools, it was established that the single nucleotide polymorphism (SNP) *ESRA* (OMIM* *133430) V364E* (rs121913044; 1453T>A; Val364Glu) generates a deleterious change with possible damage to the N-terminus of the hormone-binding domain in the ER, giving rise to a 40 times lower affinity for oestrogen (Flicek et al., 2011). This SNP also exhibits a dependence on oestrogen for binding to an *ERE*, although it maintains its negative dominant activity entirely. Therefore, *ESRA* V364E is highly active and capable of repressing ER-mediated transcription, both when *ESRA* V364E and normal ESRA proteins are present together in cells and even without DNA binding (Wrenn and Katzenellenbogen 1993; Ince et al., 1995; McInerney et al., 1996).

Although in the last decade several studies have investigated genetic variants in TAM-metabolizing enzymes that might determine the differences in the response to treatment in patients with BC, there are still controversies about their relationship with the response (Brauch and Schwab, 2014; Brewer et al., 2014; Province et al., 2014; Binkhorst et al., 2015). To address this question, we aimed to study the association among response (relapse and ADR) and 7 genetic variants in genes encoding proteins involved in the pharmacokinetics and pharmacodynamics of TAM (*CYP2D6∗4, CYP3A4∗1B (CYP3A4∗1.001), CYP3A5∗3, SULT1A1∗2, UGT2B7∗2, UGT2B15∗2* and *ESRA* V364E) in women with hormone-dependent BC and under adjuvant treatment with TAM. The main goal is to generate predictive models to approximate the response of patients according to their genetic-metabolic characteristics.

## 2 Patients and Methods

### 2.1 Study Design

A retrospective case-control study was carried out from January 2014 to January 2015 at the Polyclinic of Oncology of the National Institute of Cancer (INC). The sample size was determined according to the frequency of carriers with the variant allele carriers in the population under study, using PS Power and Sample Size Calculations Version 3.0, January 2009, considering an 80% power; α = 0.05; OR = 2.0; and the less frequent *CYP3A4∗1B* (*CYP3A4∗1.001*, rs2740574), according to the literature (Fuentes and Silveyra, 2019).

### 2.2 Patients

One hundred sixty-two (162) patients were enrolled. The inclusion criteria were as follows: 1) patients with histologically confirmed BC, 2) in adjuvant treatment with 20 mg of tamoxifen (Novadex®) daily for at least 24 months; 2) > 18 years-old, 3) positive ER and negative HER-2 status, 4) cell differentiation degree I to III. The exclusion criteria were as follows: 1) patients diagnosed with *in situ* BC; 2) negative ER, negative PR, triple-negative or Her-2 positive patients; 3) concomitant treatments with vitamin K antagonists (warfarin, acenocoumarol or dicoumarol), serotonin reuptake antagonist antidepressants (fluoxetine or paroxetine), mitomycin, nitonavir, primidone, or chemotherapy (fluorouracil, methotrexate or cyclophosphamide), 4) chronic unbalanced systemic pathology or other active cancers and, 5) aromatase inhibitor treatment or LHRH agonist. The events (relapses and ADR) were evaluated after 6 months of TAM treatment. All the patients signed a written consent form and agreed to be included in this study.

Toxicity was assessed based on the Common Terminology Criteria for Adverse Events, v4.03. Vaginal bleeding was defined as any vaginal bleeding in postmenopausal patients (more than 1 year without menstruation or oophorectomized) or any abnormal bleeding, such as unexpected bleeding or in quantity or habitual bleeding in premenopausal patients. Endometrial hyperplasia was defined as an endometrial thickness greater than 5 mm detected by transvaginal echotomography in postmenopausal patients or an abnormal thickness of the endometrium according to the phase of the menstrual cycle in premenopausal patients.

The ethnicity of the study group was approached by the percentage of Amerindian-Caucasian admixture (%M_a-c_) based on the ABO system (Valenzuela et al., 1987; Acuña et al., 2000).

The patients were gathered into two groups: the control group include patients who do not present the analyzed ADRs (relapse, hot flashes, etc.), and the cases group include patients who have the ADRs.

### 2.3 Genotyping Analyses

Potentially functional SNPs encoding the proteins related to the TAM response were obtained from the PharmGKB database (Whirl-Carrillo et al., 2012), the NCBI dbSNP database (https://www.ncbi.nlm.nih.gov/snp/), the SNPinfo Web Server (https://snpinfo.niehs.nih.gov), the Ensembl® genome database project (https://www.ensembl.org/index.html) and the level of evidence for each SNP (Supplementary Table S1). Genomic DNA was isolated from the peripheral blood samples of the subjects using a Genomic DNA Extraction Blood DNA Kit (FAVORGEN®, BIOTECH CORP, Headquarters, Taiwan, China) and from buccal mucosa cells using a MasterAmp™ Buccal Swab Kit (Epicentre®, an Illumina company, Madison, USA). DNA samples were quantified at 260/280 nm using a Nanodrop spectrophotometer (model DS-11, FX Series Spectrophotometer, USA). *CYP2D6∗4* (rs3892097), *CYP3A4∗1B* (*CYP3A4∗1.001*, rs2740574), *CYP3A5∗3* (rs776746), *SULT1A1∗2* (rs9282861), *UGT2B7∗2* (rs7439366), *UGT2B15∗2* (rs1902023), and *ESRA* V364E (rs121913044) were analyzed using polymerase chain reaction-restriction fragment length polymorphism (PCR-RFLP) (Cavalli et al., 2001; Schur et al., 2001; Hajdinjak and Zagradišnik 2004; Lee et al., 2005; Kagaya et al., 2007; Arslan et al., 2011). The primers and restriction enzymes used are presented in Supplementary Table S2. Each assay contained four controls: one sample for each genotype as a positive control and one negative sample with nuclease-free pure water to volume. For quality assurance purposes, we randomly chose 20% of the samples for repetition of the analysis. When analyses were not coincident, we excluded the samples.

### 2.4 Statistical Analyses

Statistical analyses were performed using GraphPad Prism 4.0 and STATA 11.1, with *p* < 0.05 considered statistically significant. The results are expressed as the mean ± standard deviation (SD), number, percentage, or frequency where appropriate. The Shapiro-Wilk test was used to determine quantitative variable distributions. Mean values between the two groups were compared using the unpaired t-test for variables with a normal distribution, while the Mann-Whitney U test was used for variables with non-normal distributions. Frequencies of qualitative variables were also calculated. The chi-square test with Fischer’s exact test was used to investigate differences in genotypic and allelic frequencies between the two groups.

We checked for Hardy-Weinberg equilibrium (HWE) in our sample even though the conditions for HWE are not applicable because it is not a random sampling in a random-mating population, a control population or the general population (Namipashaki et al., 2015) and is a group with a selection bias based on the disease (i.e., SNPs can also be related to cancer). The polymorphic variants *CYPD6*∗4, *CYP3A4∗1B* (*CYP3A4∗1.001*), *CYP3A5*∗3, *SULT1A1*∗2, and accomplish HWE, while *UGT2B15*∗2 and *ESRA V364E* do not (cut-off chi^2^: 3.84).

The polymorphisms were evaluated using co-dominant (wild type vs heterozygote vs variant), dominant (wild type vs. heterozygote/variant), and recessive (wild type/heterozygote vs. variant) inheritance models. Bi-variable analyses were performed to determine the association between the events (relapses and ADR), considered the dependent variables, and the characteristics of the patients, considered the independent variables. Thus, the independent variables considered were BMI, blood group, smoking habit, socioeconomic status, treatment with oral contraceptives, hormone replacement therapies, age at menarche, menopausal status, family history of cancer, age at diagnosis, cancer stage, and presence of genetic variants (exposure variable).

Multivariate logistic regression models and multivariate linear regression analyses were employed to investigate associations between genetic or non-genetic characteristics and relapse or ADR. The logistic multivariate models were adjusted stepwise using a forward and backward procedure to include potentially relevant variables to derive statistical association models characterized by pseudo R^2^. All association studies were assayed by choosing parameters with a better statistical association for each analysis. The odds ratio (OR), or coefficients (Coef.), and 95% confidence intervals (CIs) are reported in the multivariate logistic regression models.

### 2.5 Ethics

The study was carried out under strict ethical procedures recommended by the Ethics Committee of the Faculty of Medicine of the University of Chile (July 24, 2013) and the Research Ethics Committee of the Northern Metropolitan Health Service (June 3, 2013), in accordance with the procedures suggested in the Declaration of Helsinki (WMA, 2013) and according to Chilean Laws 20.120, 20.584, and 19.628 and the guidelines of Good Clinical Practices. The treatment schedule for patients was according to the cancer stage (I-III), histology, cell differentiation degree, and presence and relative abundance of 3 selected differentially abundant proteins (ER+, PR+, Her2-), which involved surgery followed by radiotherapy and/or chemotherapy according to the Breast Cancer Clinical Guide, 2nd Ed. Santiago, Chile.

## 3 Results

The baseline characteristics of the patients (58 ± 13 years; BMI: 29 ± 6) are shown in Table 1, and the characteristics of the patients according to genotype are shown in Supplementary Table S3. Sociogenetic gradients, risk factors, gynecological characteristics, and pathological features for descriptive analyses can also be observed. Of the total patients, 68 were premenopausal, and 94 were postmenopausal. In relation to the cancer stage at diagnosis, the majority of patients were in stage II (51.23%), the predominant histology type was invasive ductal carcinoma (86.88%), and the cell differentiation degree was predominantly G2 (54.17%). A 49.3% M_a-c_ was found.

**TABLE 1:** Characteristics of patients (*n* = 162).

Variables	N*	(%)	x̄ ± SD
Anthropometric characteristics			
Age (years)	160	(98.77)	58 ± 13
Weight, (Kg)	160	(98.77)	69 ± 15
Height, (m)	156	(96.30)	1.55 ± 0.06
BMI (Kg/m^2^)	156	(96.30)	29 ± 6
Socio-genetic gradient			
Blood type			
AB	3	(1.85)	
A	21	(12.96)	
B	8	(4.94)	
O	66	(40,74)	
N.D	64	(39.51)	
Number of members in the family			3 ± 2
Socioeconomic (income/member)			
<$CLP135,000 (U$ 200)	33	(21.15)	
$CLP135,001–500,000 (U$ 200–750)	91	(58.33)	
$CLP 500,001–1,000,000 (U$ >750–1,450)	27	(17.31)	
>$CLP 2,000,000 (U$ >2,900)	5	(3.21)	
N.D	6	(3.85)	
Risk factor’s			
Alcoholic Habit Presence	0	(100)	
Presence of Smoking Habit	48	(30.19)	
Presence family history of some cancer	100	(62.89)	
Presence Family History of breast or ovary cancer	43	(27.04)	
Gynecological Characteristics			
Menarche age (years)	153	(94.44)	13 ± 2
Number of Gestations	160	(98.77)	3 ± 2
Number of deliveries	160	(98.77)	3 ± 2
Number of Abortions	160	(98.77)	1 ± 1
Breastfeeding time (months)	137	(84.57)	24 ± 28
Oral Contraceptive Treatment (months)	154	(95.06)	39 ± 69
Menopausal status	68	(41.98)	
Premenopause			
Postmenopause	94	(58.02)	
Treatment with HRT (months)	21	(22,34)	11 ± 46
Pathological Features			
Age of diagnosis (years)	160	(98.77)	54 ± 13
Cancer stage at diagnosis			
I	59	(36.42)	
II	83	(51.23)	
III	20	(12.35)	
Tumor Histology			
*In situ* Ductal carcinoma (DCis)	4	(2.47)	
Invasive Ductal Carcinoma (IDC)	139	(85.80)	
Invasive Lobular Carcinoma (ILC)	8	(4.94)	
Others, (IBC, IPC, etc.)	9	(5.56)	
N.D.	2	(1.23)	
Cell Differentiation Degree			
G1	41	(25.31)	
G2	78	(48.15)	
G3	25	(15.43)	
N.D.	18	(11.11)	
Treatment before TAM			
Surgery	12	(7.41)	
Surgery + radiotherapy	37	(22.84)	
Surgery + chemotherapy	13	(8.02)	
Surgery + chemotherapy + radiotherapy	36	(22.22)	
No treatment	64	(39.51)	

N.D: no data; TAM: tamoxifen; SD: standard deviation; IBC: inflammatory breast cancer; IPC: intracystic papillary carcinoma; HRT: hormone replacement therapy.

a some data for patients were lost from the clinical files, so the numbers are lower than the total.

Table 2 shows the therapeutic response characteristics of the patients. Relapse was found in 9 patients (5.73%). The most severely observed ADRs among patients were endometrial hyperplasia (9.20%) and vaginal bleeding (4.91%), and the most frequent were hot flashes (61.96%), bone pain (13.50%), and cramps (12.27%).

**TABLE 2:** Relapse and Adverse drug reactions (ADRs) in patients.

Clinical response		
	N	(%)
Relapse		
No	148	(91.36)
Yes	9	(5.56)
N.D.	5	(3.09)
ADRs		
Endometrial cancer		
No	161	(99.38)
Yes	1	(0.62)
Endometrial hyperplasia		
No	147	(90.74)
Yes	15	(9.26)
Vaginal bleeding		
No	154	(95.06)
Yes	8	(4.94)
Phlebitis		
No	160	(98.77)
Yes	2	(1.23)
Headache		
No	157	(96.91)
Yes	5	(3.09)
Nausea		
No	156	(96.30)
Yes	6	(3.70)
Hot flash		
No	62	(38.27)
Yes	100	(61.73)
Cramps		
No	142	(87.65)
Yes	20	(12.35)
Bone pain		
No	140	(86.42)
Yes	22	(13.58)
Urticaria		
No	158	(97.53)
Yes	4	(2.47)

ADR, adverse drug reaction, evaluated with Common Terminology Criteria for Adverse Events [CTCAE], 2010. N.D: No data. Control groups include patients who do not present the analyzed ADRs (relapse, hot flashes, etc., i.e. “No”), and Cases groups include patients who have the ADRs (i.e. “Yes”).

The genotypic and allelic frequencies for the analyzed polymorphisms are shown in Table 3.

**TABLE 3:** Genotype and allele frequencies of the studied polymorphisms in patients.

Polymorphisms	N Frequency	
Enzymes involved in the activation of TAM		
*CYP2D6*		
*1/*1 (GG)	121	(0.747)
*1/*4 (GA)	37	(0.228)
*4/*4 (AA)	4	(0.025)
*1 (G)	279	(0.861)
*4 (A)	45	(0.139)
*CYP3A4		
*1/*1 (AA)	144	(0.889)
*1/*1B (AG)	17	(0.105)
*1B/*1B (GG)	1	(0.006)
*1 (A)	305	(0.941)
*1B (G)	19	(0.059)
*CYP3A5*		
*1/*1 (AA)	5	(0.031)
*1/*3 (AG)	59	(0.364)
*3/*3 (GG)	98	(0.605)
*1 (A)	69	(0.213)
*3 (G)	255	(0.787)
Enzymes involved in the elimination of TAM and its metabolites		
*SULT1A1*		
*1/*1 (GG)	33	(0.204)
*1/*2 (GA)	88	(0.543)
*2/*2 (AA)	41	(0.253)
*1 (G)	154	(0.475)
*2 (A)	170	(0.525)
*UGT2B7*		
*1/*1 (TT)	19	(0.117)
*1/*2 (TC)	72	(0.444)
*2/*2 (CC)	71	(0.438)
*1 (T)	110	(0.340)
*2 (C)	214	(0.660)
*UGT2B15*		
*1/*1 (AA)	20	(0.123)
*1/*2 (AC)	94	(0.580)
*2/*2 (CC)	48	(0.296)
*1 (A)	134	(0.414)
*2 (C)	190	(0.586)
Estrogen receptor. TAM therapeutic target		
*ESRA* V364E		
364V/364V (TT)	101	(0.623)
364V/364E (TA)	33	(0.204)
364E/364E (AA)	28	(0.173)
364V (T)	235	(0.725)
364E (A)	89	(0.275)

TAM: tamoxifen; CYP: Cytochrome P450; SULT: sulfotransferase; UGT: Uridine 5′-diphospho-Glucuronosyltransferase, ESR: estrogen receptor.

*CYP3A4*1B is currently CYP3A4*1.001 according to PharmGKB (pharmgkb.org).

We performed univariable logistic regression of the risk of relapse (Supplementary Tables S4, S5), endometrial hyperplasia (Supplementary Tables S6, S7), and vaginal bleeding (Supplementary Tables S8, S9) in association with genetic and non-genetic variables. In Table 4, only statistically significant results for the univariable logistic regression analysis (risk of relapse, endometrial hyperplasia, and vaginal bleeding) are shown. The results show that *UGT2B15∗2* A/C (rs1902023) in a dominant model of inheritance was associated with relapse*; CYP3A5∗3* A/G (rs776746) in a recessive model of inheritance was associated with endometrial hyperplasia; and *SULT1A1∗2* G/A (rs9282861) in a dominant model of inheritance and *ESRA* V364E (rs121913044) in a recessive model of inheritance were associated with vaginal bleeding.

**TABLE 4:** Univariable logistic regression analysis of risk of severe ADRs according to genotypes.

	Cases	Controls	OR**	95%CI***	*p-value* ^*#*^
Efficacy					
Relapse					
*UGT2B15*					
*1/*1 (AA)	4	15	Ref	—	—
*1/*2 (AC)	2	85	0.088	0.015–0.525	**0.008**
*2/*2 (CC)	3	40	0.281	0.056–1.407	0.123
*1/*1 (AA)	4	16	Ref		
*2/*2 (CC) + *1/*2 (AC)	5	125	0.160	0.031–0.910	**0.019**
*1/*1 (AA) + *1/*2 (AC)	6	100	Ref		
*2/*2 (CC)	3	40	1.305	0.193–6.188	0.509
Safety					
Endometrial hyperplasia					
*CYP3A5*					
*1/*1 (AA)	2	3	Ref.	—	—
*1/*3 (AG)	9	48	0.281	0.041–1.930	0.197
*3/*3 (GG)	4	89	0.067	0.009–0.524	**0.010**
*1/*1 (AA)	2	3	Ref.		
*3/*3 (GG) + *1/*3 (AG)	13	137	0.142	0.015–1.891	0.074
*1/*1 (AA) + *1/*3 (AG)	11	51	Ref.		
*3/*3 (GG)	4	89	0.208	0.047–0.758	**0.007**
Vaginal bleeding					
*SULT1A1*					
*1/*1 (GG)	4	28	Ref.	—	—
*1/*2 (GA)	1	81	0.086	0.009–0.806	**0.032**
*2/*2 (AA)	3	38	0.552	0.115–2.668	0.460
*1/*1 (GG)	4	28	Ref.		
*2/*2 (AA) + *1/*2 (GA)	4	119	0.235	0.042–1.361	0.057
*1/*1 (GG) + *1/*2 (GA)	5	109	Ref.		
*2/*2 (AA)	3	38	1.668	0.254–9.300	0.357
*ESRA V364E*					
364V/364V (TT)	3	96	Ref.	—	—
364V/364E (TA)	1	29	1.10	0.111–11.017	0.933
364E/364E (AA)	4	22	5.81	1.214–27.882	**0.028**
364V/364V (TT)	3	96	Ref.	—	—
364E/364E (AA) + 364V/364E (TA)	5	51	3.137	0.579–20.848	0.114
364V/364V (TT) + 364V/364E (TA)	4	125	Ref.	—	—
364E/364E (AA)	4	22	5.68	0.966–32.397	**0.028**

*ADR, adverse drug reaction, evaluated with CTCAE4.03; **OR, odds ratio; ***95% CI, 95% confidence interval; ^#^Logistic regression. Only statistically significant associations are shown (*p* < 0.05).

**FIGURE 1:**
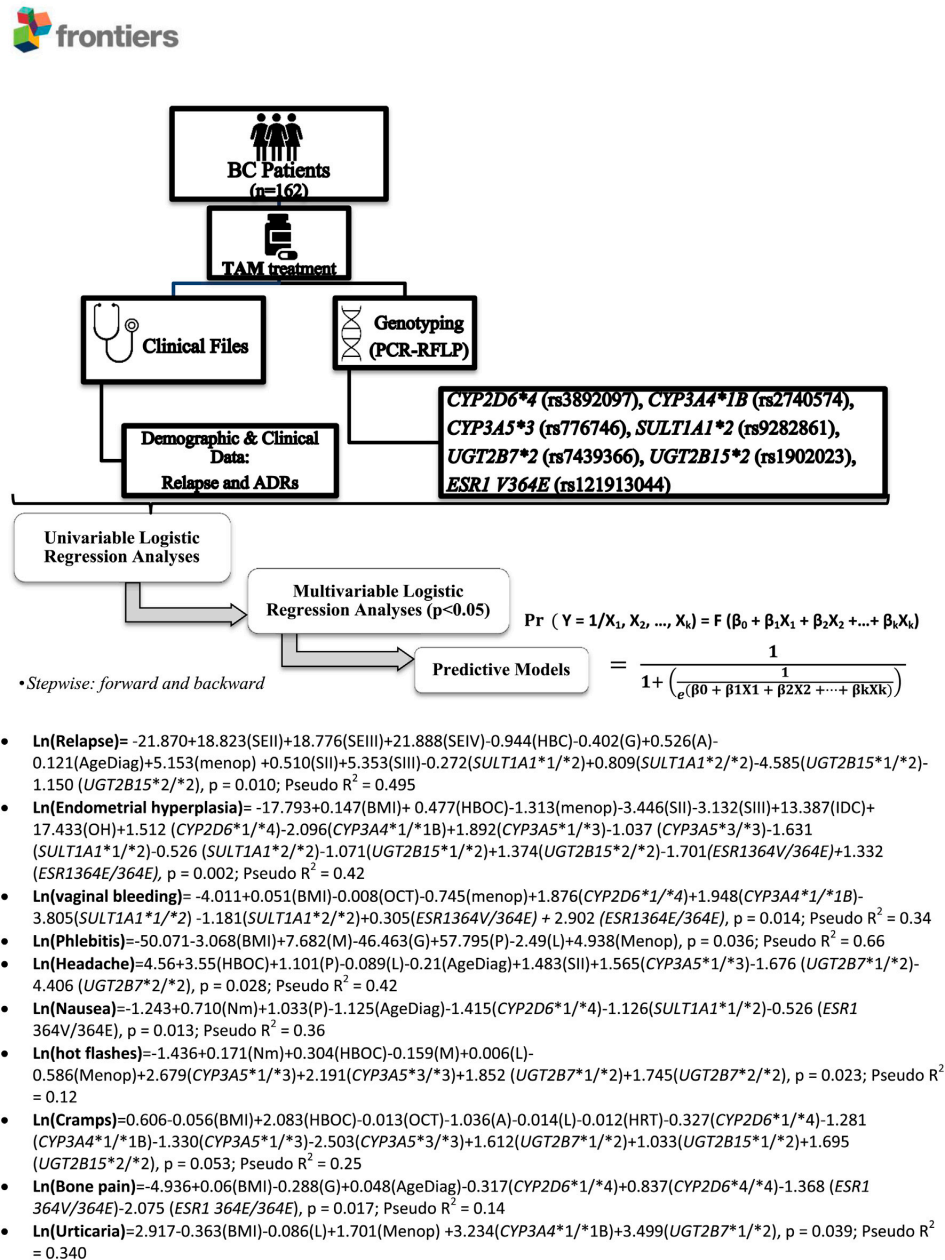
Research Scheme and obtained multivariate predictive models. ADRs, adverse drug reactions; BC: Breast Cancer; SULT: Sulfotransferase; UGT: UDP-glucuronosyl transferase; CYP: Cytochrome P-450; ESRA: Estrogen Receptor 1; SE: Socioeconomic status (per capita income), I: <$135.000 CLP (U$ 200); II: $135.001-$500.000 CLP (U$ 200–750); III: $500.001-$1.000.000 CLP (U$ >750–1,450); IV: >2.000.000 CLP >$CLP 2,000,000 (U$ >2,900); HBC: Family History of Breast Cancer; HBOC: Family History of breast or ovary cancer; M: Menarche age; G: Number of Gestations; A: Number of Abortions; P: Number of deliveries; L: Breastfeeding time; Age diag: Age of diagnosis, years; OCT: Oral Contraceptive Treatment; HRT: Treatment with HRT for menopause; Menop: postmenopause; S: Cancer stage at diagnosis (Stage I-IV); DCis: Ductal carcinoma in situ; IDC: Invasive Ductal Carcinoma; ILC: Invasive Lobular Carcinoma; OH: Others Histology (OH)*.*

After using stepwise forward and backward procedures, multivariate logistic regression analyses for the risk of relapse and ADRs, including genetic and non-genetic variables, were performed. We obtained significant models for endometrial hyperplasia, vaginal bleeding, phlebitis, headache, nausea, hot flash, cramps, bone pain, and urticaria (Figure 1). Supplementary Tables S10–S12 show significant multivariate logistic regression analyses or logit models for relapse (Supplementary Table S10), endometrial hyperplasia (Supplementary Table S11) and vaginal bleeding (Supplementary Table S12) with Pseudo R^2^ = 0.495 (*p* = 0.01), Pseudo R^2^ = 0.42 (*p* = 0.002) and Pseudo R^2^ = 0.34 (*p* = 0.014), respectively.

Associations among genetic variants with any adverse effect *versus* no adverse effect or with some grades of adverse effects were also assessed, but no statistical significance was observed (Supplementary Tables S13, S14).

## 4 Discussion

Patient response to TAM has been investigated for a long time. In this respect, differences of approximately 25–50% in patients are observed (Fisher et al., 1998; Davies et al., 2011). This is an important issue due to the harmful effects on bone metabolism, cancer of the endometrium, thromboembolic diseases, and cognitive disorders caused by chronic oestrogen deprivation in brain tissue (Fisher et al., 1998; Cuzick et al., 2002; Cuzick, 2005). Moreover, various side effects similar to those seen in menopause may occur, such as hot flashes, weight gain, vaginal dryness, nausea, and elevated liver transaminases (Cuzick, 2005; Carpenter and Miller 2005).

Although it is well known that genetic variants in *CYP2D6* could explain between 10–20% of these cases and its use as a biomarker for TAM is recommended by the FDA, there is still no agreement on its clinical utility for predicting results in BC, the studies reveal contradictory results and the role of other pharmacokinetic and pharmacodynamic proteins have been poorly studied (Brauch and Schwab, 2014; Brewer et al., 2014; Province et al., 2014; Binkhorst et al., 2015; Goetz et al., 2018). Therefore, the present research focused on studying the association between 7 genetic variants in several genes encoding proteins involved in the pharmacokinetics and pharmacodynamics of TAM and response (relapse) and ADRs in TAM-treated hormone-dependent BC women and contributing to the field, generating predictive models that approximate the response of patients according to their genetic-metabolic characteristics. CYP2D6∗3 and ∗17 alleles were previously found to be absent in different sub-groups of the Chilean population (Roco et al., 2012; Varela et al., 2015) therefore we exclude them from this study and other potentially relevant variants could not be studied, unfortunately.

In this study, we recorded ADRs after 6 months of TAM treatment. This is because several other factors influence TAM ADRs, especially before TAM active metabolites reach steady state, which is after 2 months of treatment (Buckley and Goa, 1989; Lien et al., 1992; Kisanga et al., 2004). Therefore, we considered that after 6 months of treatment, the ADRs might be associated mainly with TAM. Similarly, after 2 years of treatment, relapses can be properly evaluated (Cheng et al., 2012).

The study patients have a 49.3% M_a-c_, which agrees with that described by Valenzuela *et al.* in 1987, in relation to admixture and socioeconomic stratum, because main of the enrolled population of patients (94%) belong to a low socioeconomic stratum with higher Mapuche ancestry; this was an expected result according to the place of recruitment of the patients.

The study of the association between genetic variants and clinical parameters showed that the presence of *UGT2B15*∗1/∗2 protects against relapse (OR = 0.09; *p* = 0.008), *CYP3A5∗3/∗3* protects against presenting endometrial hyperplasia (OR = 0.07, *p* = 0.01), and *SULT1A1∗1/∗2* protects against vaginal bleeding (OR = 0.09; *p* = 0.03) and that *ESRA* 364E/364E is a risk factor for vaginal bleeding (OR = 5.68; *p* = 0.03). In this respect, it seems to be relevant to perform more studies regarding the *ESRA* V364E variant, a very poorly studied variant that can be determined in patients with ER + BC to prevent vaginal bleeding caused by TAM therapy, which is also a suspicious sign of endometrial cancer.

In our study, some analyses showed associations without statistical significance, which may be due to the low number of occurrences of the event in each subgroup, lack of clinical data in any of the contrasting groups, or because the variant explains a smaller part of the response, which could be clarified by increasing the sample size in future studies. Thus, in small groups, Fisher’s exact test was used to obtain proper results.

The biostatistical tools used in this field have established that non-significant trends, but with *p* < 0.2 and physiological significance, can be included in predictive models and in multi-variable logistic regression analysis to help predict the response (relapses) and adverse reactions to treatment (Núñez et al., 2011). Therefore, preliminary predictive models to predict the response (efficacy and safety) in TAM-treated BC patients were generated. The efficacy result, analyzed as relapse, led to a model that explains 49.5% of the possibility of presenting a relapse (*p* = 0.01) by including *SULT1A1∗2* and *UGT2B15∗2* and a series of relevant non-genomic variables. These results were expected since both the SULT1A1 and UGT2B15 enzymes have specificity for 4-hydroxyTAM, even though the genetic variant *UGT2B15∗2* presents a nucleotide change located in a substrate binding site that is associated with a decrease in catalytic activity and alterations in the Km and Vmax values (Lévesque et al., 1997). Similarly, *SULT1A1∗2* has a lower catalytic activity than the wild-type allele (Arslan et al., 2011). Thus, these variants would produce a decrease in the elimination of 4-hydroxyTAM.

Moreover, our results are similar to those published by Nowell and his colleagues (Nowell et al., 2002 and Nowell et al., 2005), who conducted a retrospective study in 162 TAM-treated BC patients from Arkansas who received TAM to determine whether genetic variability (*CYP2D6∗4, SULT1A1∗2,* and *UGT2B15∗2*) in the TAM metabolic pathway was associated with recurrence. They found that high activity genotypes *UGT2B15 ∗1/∗1* and combined genotypes *UGT2B15∗1/∗1* and *SULT1A1∗2/∗2* lead to an increased risk of disease recurrence and that *SULT1A1∗2/∗2* causes an approximately 3 times higher risk of death than *SULT1A1∗1/∗1* and *∗1/∗2*. Conversely, Wegman et al. (2005) found that the genetic variants *CYP2D6∗4/∗4,* and *SULT1A1∗1/∗1* cause an increase in the disease-free relapse time in patients with premenopausal and postmenopausal BC treated with 40 mg TAM using proportional hazards Cox regression adjusted for age, tumor size, and lymph nodes. However, Ahern et al. (2011) and Lash et al. (2011) in homologous models found that the genetic variants *CYP2D6∗4, UGT1A8∗3, UGT2B7∗2,* and *UGT2B15∗2* did not modify the relapse rate of the disease in patients with ER+/TAM + BC. On the other hand, Sensorn et al., 2013 investigated genetic variant *CYP3A4∗1B* (−392A>G, actually CYP3A4∗1.001 G/A)/∗18 (878T>C), CYP3A5∗3 (6986 G>A), *ABCB1 3435 C>T*, *ABCC2∗1C* (−24C > T) and *ABCC2 68231 A>G* to evaluate the risk of recurrence within 3 years among thirty Thai women after receiving tamoxifen adjuvant therapy but they could not find *CYP3A4∗1B/∗18*, and did not observe a statistical association between *CYP3A5∗3, ABCC2∗1C*, and *ABCC2 68231 A>G* with clinical outcome.

Additionally, preliminary predictive models were generated to predict ADRs. The endometrial hyperplasia model explained 41.6% of the possibility of presenting the event (*p* = 0.002). The model includes the *CYP2D6∗4, CYP3A4∗1B (CYP3A4∗1.001), CYP3A5∗3, SULT1A1∗2, UGT2B15∗2* and *ESRA* V364E genotypes plus several relevant non-genomic factors. Likewise, a preliminary predictive model was generated that explains 33.7% of the possibility of presenting vaginal bleeding (*p* = 0.014) by including the *CYP2D6∗4, CYP3A4∗1B (CYP3A4∗1.001), SULT1A1∗2,* and *ESRA* V364E genotypes and relevant non-genomic factors. Currently, there is no evidence regarding the association of genetic variants with TAM-induced vaginal bleeding or endometrial thickening. Chu et al. (2007) found that women with BC who carried *CYP3A4∗1B (CYP3A4∗1.001),* had a 3-fold increased risk of developing endometrial cancer after treatment with TAM. The phlebitis model explained 66% of the possibility of presenting this ADR (*p* = 0.036), a significant finding that has not been previously reported. In the same way, preliminary predictive models were generated to predict the appearance of headache (42.2%; *p* = 0.028), nausea (36.3%; *p* = 0.013), and hot flashes (12.1%; *p* = 0.023) in patients. Our results differ from those reported by Goetz et al. (2005), where *CYP2D6∗4/∗4* tends to contribute to a higher risk of disease relapse and a lower incidence of hot flashes in a similar study. No other studies were found in the literature describing associations with headache and nausea. Finally, predictive models to predict the appearance of cramps (24.6%; *p* = 0.053), bone pain (14.1%; *p* = 0.017) and urticaria (34%; *p* = 0.039) were obtained. We were not able to find any similar study in the scientific literature.

Altogether, our results show that some specific genetic variants in genes encoding proteins involved in the TAM pharmacokinetic and/or pharmacodynamic can influence the efficacy of TAM therapy in BC.

Since there are still discrepancies between the findings, which may be due to the genetic differences present in the subjects analyzed, it is crucial to continue with similar and larger studies that confirm the association of *CYP2D6∗4, CYP2C9∗2* and *∗3, CYP3A4∗1B (CYP3A4∗1.001), CYP3A5∗3, SULT1A1∗2, UGT2B15∗2* and *ESR1* V364E and response to treatment with TAM. Differences in allele frequencies of variants influencing drug’s pharmacokinetics across populations are considered to result in the interethnic differences in pharmacokinetics observed. For example, the lower mean body weights of populations of East Asian in relation to European populations are well established to contribute to differences in the clearance or volume of distribution of some drugs (Lu et al., 2018). On the other hand, in relation to that scarce information is available in Latin American populations, but allele frequencies heterogeneously differ from Asian, Caucasian and African populations (Mendoza et al., 2001; López et al., 2005; Roco et al., 2012; Friedrich et al., 2014), giving rise an idea of the phenotypic response and patient outcome.

Despite our analysis, the present study has some limitations. Even though we had a good sample size for combinatorial analyses, a small number of patients examined in each sub-group comparison could mask potential associations, especially for low-frequency polymorphisms, particularly in the multivariate analyses. The small number of variants analyzed could restrict the statistical power and predictive potential of models, considering that some other candidate genes/polymorphisms were not evaluated in this study (e.g. *CYP2D6 ∗2, ∗5, ∗6, ∗10* and *∗41*, *CYP2C9∗2, CYP2C19 ∗2* and *∗17, UGT1A10 139Lys, UGT1A8* 173Gly/277Cys and *ABCC2*), which could still be relevant. In some cases, missing clinical data was relevant, giving rise to a possible differential misclassification bias affecting the estimated associations between potentially relevant combinations of risk factors and adverse reactions. Other environmental factors, such as contaminants and stress, or social determinants were not explored in this study, which could potentially affect the associations.

However, our findings and preliminary predictive models proposed can be used to prevent, in a good percentage of patients, the negative effects of TAM therapy. Additionally, they could help in the decision-making of a change in therapy to aromatase inhibitors (AIs) (Bernhard et al., 2015).

Certainly, for routine clinical application, the models must be validated in terms of their specificity, sensitivity, and predictive value, so it is necessary to carry out a second stage of research that involves treatment intervention in the patient according to their genotypic profile and relevant non-genomic variables derived from this study. It should be considered that the predictive models generated in this study after being validated could be used in the Chilean population. They could be used in another population with the same percentage of Caucasian aboriginal mix.

## 5 Conclusion

In our study, UGT2B15*2 A/C is likely to have a clinically significant impact on recurrence risk, *CYP3A5∗3* A/G on endometrial hyperplasia, and *SULT1A1∗2* G/A, and *ESRA* V364E on vaginal bleeding in Chilean patients with breast cancer who receive tamoxifen adjuvant therapy. The obtained predictive models showed that the response to TAM treatment in BC patients is partly associated with some of the genetic variants studied. This, after a clinical validation protocol, might help to predict a percentage of disease relapse and negative effects of TAM therapy, improving the efficacy and safety of TAM pharmacotherapeutic treatment.

## Data Availability

The raw data supporting the conclusions of this article will be made available by the authors, without undue reservation, to any qualified researcher.
