# Supplementary material for: Preliminary Pharmacogenomic-Based Predictive Models of Tamoxifen Response in Hormone-dependent Chilean Breast Cancer Patients
Source: Front Pharmacol. 2021 Nov 25;12:661443. doi: 10.3389/fphar.2021.661443 (PMC8656167; doi:10.3389/fphar.2021.661443)
Supplement: Supplementary file 1 [file DataSheet1.pdf]

Supplementary Tables

Table S1: Incidence of SNPs in other populations

Variants (rs)	change nt/aa	Effect in prot./enzyme	Africans	Asian	Caucasians	Chileans	European	Hispanics	Mapuches
CYP2D6*4 **(rs3892097)**	1846G>A	Non-functional protein[1]	0.06 (n=48)[8]	0.00 (n=256)[14]	0.24 (n=62)[11]	0.12 (n=253)[10]	ND	0.06 (n=46)[11]	0.036 (n=84)[15]
CYP3A4*1B **(rs2740574)**	c.-392A>G	↓expression[2]	0.5 (n=44)[9]	0.00 (n=80)[9]	0.04 (n=116)[9]	0.06 (n=253)[10]	0.02 (n=44)[8]	0.21 (n=211)[9]	ND
CYP3A5*3 **(rs776746)**	6986A>G	Truncated, non-functional protein, splicing defect[3]	0.15 (n=120)[8]	0.75 (n=68)[12]	0.97 (n=62)[12]	0.76 (n=253)[10]	0.96 (n=222)[8]	0.77 (n=200)[12]	ND
SULT1A1*2 **(rs928286)**	c.638G>A Arg213His	↓activity; ↓estability[4]	0.17 (n=48)[8]	ND	0.26 (n=48)[8]	ND	ND	0.24 (n=46)[8]	ND
UGT2B7*2 **(rs743936)**	c.802T>C Tyr268His	↑activity[5]	0.99 (n=120)[8]	0.25 (n=91)[13]	0.25 (n=91)[13]	0.40 (n=253)[10]	0.5 (n=120)[8]	ND	ND
UGT2B15*2 **(rs1902023)**	c.291A>C Tyr85Asp	N.D. (↓Vmax)[6]	ND	0.57[8]	0.51 (n=3576)[8]	ND	ND	ND	ND
ESRA **V364E (rs121913044)**	c.1461T>A Val364Glu	Negative dominant inhibitor[7]	ND	ND	ND	ND	ND	ND	ND

nt: nucleotide. aa: amino acid. ND: not described. [1]: Gough et al., 1990. [2]: Cavalli, Hirata, & Hirata, 2001. [3]: Lin et al., 2002. [4]: Raftogianis, Wood, Otterness, Van Loon, & Weinshilboum, 1997. [5]: Thibaudeau et al., 2006. [6]: Lévesque et al., 1997. [7]: McInerney et al., 1996. [8]: NCBI, n.d. [9]: Paris et al., 1999. [10]: Roco et al., 2012. [11]: Restrepo et al., 2011. [12]: Roy et al., 2005. [13]: Bhasker et al., 2000. [14]: Hiratsuka et al., 2002. [15]: Muñoz et al., 1998.

**CYP3A4*1B is currently CYP3A4*1.001, according to PharmGKB (pharmgkb.org).*

Table S2: Primers and restriction enzymes used in PCR-RFLP reactions to investigate selected polymorphisms.

Polymorphism	Primer	Amplicon (bp)	Restriction enzyme	RFLP stripe pattern (bp)	Reference
*CYP2D6*4* (rs3892097)	F: 5'-GCCTTCGCCAACCACTCCG-3'<br>R: 5'-AAATCCTGCTCTTCCGAGGC-3'	*355*	*MvaI*	*1/*1: 250;105<br>*1/*4: 355; 250; 105<br>*4/*4:355	Schur *et al.*, 2001
*CYP3A4*1B* (CYP3A4*1.001, rs2740574)	F: 5'-GGAATGAGGACAGCCATAGAGACAAGGGGA-3'<br>R: 5'-CCTTTCAGCTCTGTGTTGCTCTTTGCTG-3'	*385*	*MboII*	*1/*1: 175;169<br>*1/*1B:210;175;169<br>*1B/*1B:210;175	Cavalli *et al.*, 2001
*CYP3A5*3* (rs776746)	F: 5'-CTTTAAAGAGCTCTTTTGTCTCTCA-3'<br>R: 5'-GAAGCCAGACTTTGATCATTATGTTATG-3'	*196*	*BseMII*	*1/*1: 196<br>*1/*3:196;160;36<br>*3/*3:160;36	Lee *et al.*, 2005
*SULT1A1*2* (rs928286)	F: 5'-GTTGGCTCTGCAGGGTTTCTAGGA-3'<br>R: 5'-CCCAAACCCCCTGCTGGCCAGCACCC-3'	*333*	*HaeII*	*1/*1:168;165<br>*1/*2:333;168;165<br>*2/*2:333	Arslan *et al.*, 2011
*UGT2B7*2* (rs743936)	F: 5'-TTGCCTACATTTTTGCCTACA-3'<br>R: 5'-CGTGCACATGAGTTTCTAATTG-3'.	*400*	*BseGI*	*1/*1:332;68<br>*1/*2:332;221;111;68<br>*2/*2:221;111;68	Kagaya *et al.*, 2007
*UGT2B15*2* (rs1902023)	F: 5'-CTGTGGAAAGGTGCTAGT-3'<br>R: 5'-GAATTTTCAGAAGAGAATCTTCCAGAT-3'	*215*	*Sau3AI*	*1/*1: 215<br>*1/*2:215;187<br>*2/*2:187	Hajdinjak *et al.*, 2004
ESRA V364E (rs121913044)	F: 5'-ACAAGCGCCAGAGAGATGAT-3'<br>R: 5'-CCCCACTATTTCTCCCATGA-3'	*376*	*BanI*	364V/364V:290;86<br>364V/364E:376;290;86<br>364E/364E:376	

Table S3: Characteristics of patients (n = 162) according to genotype

	Case-Control Study			*CYP2D6*4*			*CYP3A4*1B (CYP3A4*1.001)*			*CYP3A5*5*			*SULT1A1*2*			*UGT2B7*2*			*UGT2B15*3*			*ESRA* V364E		
Variables	N	(%)	x̄ ± SD	G/G	G/A	A/A	A/A	A/G	G/G	A/A	A/G	G/G	G/G	G/A	A/A	T/T	T/C	C/C	A/A	A/C	C/C	T/T	T/A	A/A
N	162	100		121	37	4	144	17	1	5	59	98	33	88	41	19	72	71	20	94	48	101	33	28
**Anthropometric Characteristics**																								
Age (years)	160	(98.77)	58 ± 13	59	57	66	59	58	83	57	58	59	57	61	56	57	58	61	56	58	62	58	60	61
Weight, (Kg)	160	(98.77)	69 ± 15	69	71	68	70	68	51	72	67	71	72	70	65	75	70	67	71	71	66	69	69	71
Height, (m)	156	(96.30)	1.55 ± 0.06	1.55	1.56	1.54	1.55	1.54	1.48	1.52	1.54	1.55	1.55	1.55	1.56	1.55	1.55	1.54	1.53	1.56	1.54	1.56	1.56	1.51
BMI (Kg/m^2)	156	(96.30)	29 ± 6	29	29	29	29	28	23	31	28	29	30	29	26	31	29	28	31	29	28	29	28	31
**Socio-genetic gradient**																								
Blood type																								
AB	3	(3.06)		2	1	-	2	1	0	0	2	1	0	2	1	1	2	0	0	2	1	2	1	0
A	21	(21.43)		15	6	-	18	3	0	1	10	10	3	14	4	2	10	9	4	12	5	16	3	2
B	8	(8.16)		8	0	-	7	1	0	0	2	6	2	4	2	0	5	3	1	5	2	6	2	0
O	66	(67.35)		50	16	-	59	6	1	3	25	38	15	36	15	7	30	29	6	39	21	37	17	12
Number of members in the family			3 ± 2	3	3	4	3	3	1	3	3	3	3	3	3	3	3	3	3	3	3	3	3	3
Socioeconomic																								
<$CLP135,000 (U$ 200)	33	(21.15)		27	7	0	32	2	0	1	17	16	10	19	5	7	11	16	3	18	13	20	3	11
$CLP135,001-500,000 (U$ 200-750)	91	(58.33)		68	22	4	82	11	1	4	31	59	16	49	29	7	46	41	11	53	20	58	23	13
$CLP 500,001-1,000,000 (U$ >750-1,450)	27	(17.31)		21	7	0	26	2	0	0	10	18	5	16	7	3	13	12	4	20	4	19	5	4
>$CLP 2,000,000 (U$ >2,900)	5	(3.21)		4	1	0	4	1	0	0	1	4	2	3	0	2	2	1	2	2	1	4	1	0
No data	6	(3.85)																						
**Risk factor's**																								
Alcoholic Habit Presence	0	(100)		0	0	0	0	0	0	0	0	0	0	0	0	0	0	0	0	0	0	0	0	0
Presence of Smoking Habit	48	(30.19)		31	14	3	45	3	0	1	17	30	6	27	15	6	23	19	6	29	13	31	10	7
Presence family history of some cancer	100	(62.89)		77	19	4	88	12	0	4	35	61	17	59	24	9	45	46	10	61	29	62	23	15
Presence Family History of breast or ovary cancer	43	(27.04)		35	6	2	39	4	0	2	14	27	9	22	12	4	22	17	5	27	11	24	12	7
**Gynecological Characteristics**																								
Menarche age (years)	153	(94.44)	13 ± 2	13	13	12	13	13	-	14	13	13	13	13	13	13	13	13	13	13	12	13	13	13
Number of Gestations	160	(98.77)	3 ± 2	3	3	2	3	2	1	5	3	3	3	3	3	3	3	3	2	3	4	3	3	4
Number of deliveries	160	(98.77)	3 ± 2	3	3	2	3	2	1	4	3	2	3	3	2	2	2	3	2	2	3	2	2	3
Number of Abortions	160	(98.77)	1 ± 1	1	1	0	1	0	0	1	1	1	1	1	1	1	0	1	0	1	1	1	1	1
Breastfeeding time (months)	137	(84.57)	24 ± 28	24	23	12	23	31	3	44	28	20	17	28	22	40	24	20	16	20	34	24	15	33
Oral Contraceptive Treatment (months)	154	(95.06)	39 ± 69	44	26	2	38	43	0	0	39	40	60	36	28	57	39	33	39	41	33	40	34	40
Menopausal status																								
Premenopause	68	(41.98)		51	16	1	61	7	0	2	26	40	17	36	15	9	35	24	11	40	17	46	12	10
Postmenopause	94	(58.02)		70	21	3	83	10	1	3	33	58	16	52	26	10	37	47	9	54	31	55	21	18
Treatment with HRT for menopause (months)	21	(14.78)	11 ± 46	14	2	0	12	1	0	0	13	11	13	11	10	0	7	18	6	8	19	12	17	2
**Pathological Features**																								
Age of diagnosis (years)	160	(98.77)	54 ± 13	55	52	62	54	56	78	53	54	55	52	57	52	53	54	55	51	54	57	52	55	58
Cancer stage at diagnosis																								
I	59	(36.42)		43	13	3	50	9	0	2	20	37	10	33	16	4	24	31	6	38	15	30	21	8
II	83	(51.23)		62	20	1	70	7	0	2	30	51	18	44	21	12	35	36	11	46	26	57	11	15
III	20	(12.35)		16	4	0	18	1	1	1	9	10	5	11	4	3	13	4	3	10	7	14	1	6
Tumor Histology																								
Ductal carcinoma in situ (DCis)	4	(2.47)		3	1	0	2	2	0	0	1	3	0	3	1	0	1	3	0	3	1	1	2	1
Invasive Ductal Carcinoma (IDC)	139	(85.80)		105	31	3	124	14	1	3	51	85	27	75	37	19	63	57	16	83	40	86	28	25
Invasive Lobular Carcinoma (ILC)	8	(4.94)		5	2	1	7	1	0	0	4	4	2	4	2	0	3	5	2	4	2	5	2	1
Others, (IBC, IPC, etc.)	9	(5.56)		6	3	0	9	0	0	2	2	5	4	4	1	0	5	4	2	4	3	7	1	1
No data	2	(1.23)																						
Cell Differentiation Degree																								
G1	41	(25.31)		29	10	2	36	5	0	0	18	23	9	26	6	7	20	14	6	23	12	21	12	8
G2	78	(48.15)		57	21	0	71	7	0	3	26	49	14	36	28	11	32	35	8	49	21	48	13	17
G3	25	(15.43)		22	2	1	20	4	1	1	7	17	7	14	4	1	11	13	2	14	9	17	5	3
No data	18	(11.11)																						
Treatment before TAM																								
Surgery	12	(7.41)		10	2	0	11	1	-	0	3	9	1	9	2	0	7	5	1	6	5	5	4	3
Surgery + radiotherapy	37	(22.84)		29	7	1	31	6	-	1	13	23	6	22	9	4	14	19	3	20	4	9	18	10
Surgery + chemotherapy	13	(8.02)		11	2	0	11	2	-	0	5	8	4	3	6	3	8	2	3	8	2	8	2	3
Surgery + chemotherapy + radiotherapy	36	(22.22)		30	6	0	33	3	-	1	13	22	6	23	7	5	16	15	5	21	10	16	8	12
No treatment	64	(39.51)																						

Table S4: Demographic aspects of patients with relapse (cases) and without relapse (controls) for the retrospective study and univariable logistic regression analysis.

Variables	Cases (n= 9)		Controls (n=148)		*p-value*	*p-value#*
**Anthropometric Characteristics**						
Age (years)	9	62 ± 15	139	59 ± 13	0.536**	0.508
Weight. (Kg)	9	71 ± 15	140	69 ± 15	0.696**	0.725
Height. (m)	9	1.55± 0.08	136	1.55 ±0.07	0.898***	0.897
BMI (Kg/m^2)	9	30 ± 7	136	29 ± 6	0.706**	0.558
**Socio-genetic gradient**						
Blood type	5		85			
AB	0		3		1.000*	0.791
A	1		19			
B	0		8			
O	4		55			
Number of members in the family	9	3 ± 2	139	3 ± 2	0.537**	0.634
Socioeconomic	9		139			
<$135.000.-	0		31		0.118*	**0.048**
$135.001-$500.000.-	7		81			
$500.001-$1.000.000.-	1		24			
>2.000.000.-	1		3			
**Risk factor's**						
Alcoholic Habit Presence	8	0	138	0	-	-
Presence of Smoking Habit	8	3	138	39	0.690*	0.583
Presence family history of some cancer	8	3	138	87	0.260*	**0.155**
Presence Family History of breast or ovary cancer	8	2	138	36	1.000*	0.945
**Gynecological Characteristics**						
Menarche age (years)	7	13 ± 4	135	13 ± 2	0.677**	0.824
Number of Gestations	9	3 ± 2	139	3 ± 3	0.657**	0.529
Number of deliveries	9	2 ± 1	139	3 ± 2	0.602**	0.414
Number of Abortions	9	1 ± 1	139	1 ± 1	0.711**	0.990
Breastfeeding time (months)	6	22 ± 17	121	25 ± 29	0.900**	0.786
Oral Contraceptive Treatment (months)	8	44 ± 63	135	36 ± 64	0.875**	0.755
Treatment with HRT for menopause (months)	9	15 ± 25	123	11 ± 49	**0.075****	0.844
**Pathological Features**						
Age of diagnosis (years)	9	54 ± 16	140	54 ± 13	0.899***	0.903
Menopausal status						
Premenopause	3		57		0.740*	0.658
Postmenopause	6		83			
Cancer stage at diagnosis	9		148			
I	2		53		0.085*	**0.151**
II	4		74			
III	3		13			
Tumor Histology	9		140			
Ductal carcinoma in situ (DCis)	0		4		1.000*	-
Invasive Ductal Carcinoma (IDC)	9		120			
Invasive Lobular Carcinoma (ILC)	0		8			
Others. (IBC. IPC. etc.)	0		8			
Cell Differentiation Degree	8		126			
G1	4		34		0.367*	0.411
G2	3		70			
G3	1		22			
Treatment before to TAM	5		84			
Surgery	1		10		0.707*	0.833
Surgery + radiotherapy	2		31			
Surgery + chemotherapy	1		11			
Surgery + chemotherapy + radiotherapy	1		32			

****Student test for parametric data; ** Mann-Whitney test for non-parametric data;* * Fisher's exact test for both categorical variable; #*Logistic regression*

Table S5.- Genotypic and allelic frequencies of *CYP2D6*4* (rs3892097), *CYP3A4*1B* (*CYP3A4*1.001*, rs2740574), *CYP3A5*3* (rs776746), *SULT1A1*2* (rs9282861), *UGT2B7*2* (rs7439366), *UGT2B15*2* (rs1902023), and *ESRA* V364E (rs121913044) polymorphisms in patients with relapse (cases) and without relapse (controls) for a retrospective study and univariate logistic regression analysis.

**Polymorphism in genes**	Cases (n= 9)			Controls (n=140)			*p-value**	$^{\$}$*HWE*	*p-value#*
Enzymes involved in the activation of TAM									
CYP2D6									
**1/*1 (G/G)*	6	(0.667)		104	(0.743)				
**1/*4 (G/A)*	3	(0.333)		33	(0.236)		0.743	YES	0.546
**4/*4 (A/A)*	0	(0.000)		3	(0.021)				
**1 (G)*	15	(0.833)		241	(0.861)				
**4 (A)*	3	(0.167)		39	(0.139)				
*****CYP3A4***									
**1/*1 (A/A)*	8	(0.889)		124	(0.886)				
**1/*1B (A/G)*	0	(0.000)		16	(0.114)		0.044	YES	-
**1B/*1B (G/G)*	1	(0.111)		0	(0.000)				
**1 (A)*	16	(0.889)		264	(0.943)				
**1B (G)*	2	(0.111)		16	(0.057)				
CYP3A5									
**1/*1 (A/A)*	0	(0.000)		5	(0.036)				
**1/*3 (A/G)*	2	(0.222)		52	(0.371)		0.624	YES	0.436
**3/*3 (G/G)*	7	(0.778)		83	(0.593)				
**1 (A)*	2	(0.111)		62	(0.221)				
**3 (G)*	16	(0.889)		218	(0.779)				
Enzymes involved in the elimination of TAM and its metabolites									
SULT1A1									
**1/*1 (G/G)*	2	(0.222)		29	(0.207)				
**1/*2 (G/A)*	5	(0.556)		73	(0.521)		1.000	YES	0.947
**2/*2 (A/A)*	2	(0.222)		38	(0.271)				
**1 (G)*	9	(0.500)		131	(0.468)				
**2 (A)*	9	(0.500)		149	(0.532)				
UGT2B7									
**1/*1 (T/T)*	2	(0.222)		16	(0.114)				
**1/*2 (T/C)*	4	(0.444)		60	(0.429)		0.494	YES	0.611
**2/*2 (C/C)*	3	(0.333)		64	(0.457)				
**1 (T)*	8	(0.444)		92	(0.329)				
**2 (C)*	10	(0.556)		188	(0.671)				
UGT2B15									
**1/*1 (A/A)*	4	(0.444)		15	(0.107)				
**1/*2 (A/C)*	2	(0.222)		85	(0.607)		0.011	NO	**0.022**
**2/*2 (C/C)*	3	(0.333)		40	(0.286)				
**1 (A)*	10	(0.556)		115	(0.411)				
**2 (C)*	8	(0.444)		165	(0.589)				
Estrogen receptor. TAM therapeutic target									
***ESRA* V364E**									
364V/364V (T/T)	8	(0.889)		85	(0.607)				
364V/364E (T/A)	0	(0.000)		30	(0.214)		0.220	NO	0.384
364E/364E (A/A)	1	(0.111)		25	(0.179)				
364V (T)	16	(0.889)		200	(0.714)				
364E (A)	2	(0.111)		80	(0.286)				

**Fisher's exact test; $^{\$}$HWE (Hardy Weinberg Equilibrium); $^{\#}$Logistic regression.*
***CYP3A4*1B is currently CYP3A4*1.001 according to PharmGKB (pharmgkb.org).*

Table S6: Demographic aspects of patients with (cases) and without (controls) endometrial hyperplasia for the retrospective study and univariate logistic regression analysis.

Variables	Cases (n= 15)		Controls (n=141)		*p-value*	*p-value#*
**Anthropometric Characteristics**						
Age (years)	15	59 ± 12	138	59 ± 13	0.933**	0.876
Weight. (Kg)	15	72 ± 15	138	69 ± 15	0.342**	0.419
Height. (m)	14	1.53 ± 0.09	135	1.55 ± 0.06	**0.254*** **	0.272
BMI (Kg/m^2)	14	31 ± 6	135	29 ± 6	**0.061****	**0.160**
**Socio-genetic gradient**						
Blood type	10		84			
AB	0		3		0.909*	0.627
A	2		19			
B	0		8			
O	8		54			
Number of members in the family	15	3 ± 2	139	3 ± 2	0.389**	0.458
Socioeconomic	15		139			
<$135.000.-	5		29		0.606*	0.512
$135.001-$500.000.-	7		83			
$500.001-$1.000.000.-	3		23			
>2.000.000.-	0		4			
**Risk factor's**						
Alcoholic Habit Presence	15	0	137	0	-	-
Presence of Smoking Habit	15	2	137	42	**0.233***	**0.133**
Presence family history of some cancer	15	9	137	85	1.000*	0.877
Presence Family History of breast or ovary cancer	15	3	137	37	0.760*	0.548
**Gynecological Characteristics**						
Menarche age (years)	14	13 ± 1	132	13 ± 2	0.823**	0.992
Number of Gestations	15	3 ± 3	138	3 ± 2	0.781**	0.789
Number of deliveries	15	3 ± 2	138	3 ± 2	0.857**	0.907
Number of Abortions	15	1 ± 2	138	1 ± 1	0.858**	0.462
Breastfeeding time (months)	14	37 ± 51	117	23 ± 24	0.846**	**0.118**
Oral Contraceptive Treatment (months)	15	27 ± 51	133	37 ± 65	0.503**	0.552
Treatment with HRT for menopause (months)	14	12 ± 36	122	12 ± 48	**0.964****	0.969
**Pathological Features**						
Age of diagnosis (years)	15	54 ± 12	142	55 ± 13	0.955***	0.815
Menopausal status						
Premenopause	6		56		1.000*	1.000
Postmenopause	9		84			
Cancer stage at diagnosis	15		140			
I	9		46		0.066*	**0.092**
II	4		77			
III	2		16			
Tumor Histology	15		139			
Ductal carcinoma in situ (DCis)	0		4		**0.013***	**0.005**
Invasive Ductal Carcinoma (IDC)	11		123			
Invasive Lobular Carcinoma (ILC)	0		8			
Others. (IBC. IPC. etc.)	4		4			
Cell Differentiation Degree	11		128			
G1	4		36		0.837*	0.679
G2	6		69			
G3	1		23			
Treatment before to TAM	9		83			
Surgery	0		12		0.427*	**0.072**
Surgery + radiotherapy	5		29			
Surgery + chemotherapy	0		12			
Surgery + chemotherapy + radiotherapy	4		30			

****Student test for parametric data; ** Mann-Whitney test for non-parametric data;* * Fisher's exact test for both categorical variable; #*Logistic regression.*

Table S7.- Genotypic and allelic frequencies of *CYP2D6*4* (rs3892097), *CYP3A4*1B* (rs2740574), *CYP3A5*3* (rs776746), *SULT1A1*2* (rs9282861), *UGT2B7*2* (rs7439366), *UGT2B15*2* (rs1902023), and *ESRA* V364E (rs121913044) polymorphisms in patients with (cases) and without (controls) endometrial hyperplasia for a retrospective study and univariate logistic regression analysis.

**Polymorphism in genes**	Cases (n=15)			Controls (n=140)			*p-value**	[$]*HWE*	*p-value#*
Enzymes involved in the activation of TAM									
CYP2D6									
**1/*1 (G/G)*	11	(0.733)		104	(0.743)				
**1/*4 (G/A)*	4	(0.267)		32	(0.229)		0.838	YES	0.789
**4/*4 (A/A)*	0	(0.000)		4	(0.029)				
**1 (G)*	26	(0.867)		240	(0.857)				
**4 (A)*	4	(0.133)		40	(0.143)				
*****CYP3A4***									
**1/*1 (A/A)*	14	(0.933)		124	(0.886)				
**1/*1B (A/G)*	1	(0.067)		15	(0.107)		1.000	YES	0.599
**1B/*1B (G/G)*	0	(0.000)		1	(0.007)				
**1 (A)*	29	(0.967)		263	(0.939)				
**1B (G)*	1	(0.033)		17	(0.061)				
CYP3A5									
**1/*1 (A/A)*	2	(0.133)		3	(0.021)				
**1/*3 (A/G)*	9	(0.600)		48	(0.343)		0.005	YES	**0.011**
**3/*3 (G/G)*	4	(0.267)		89	(0.636)				
**1 (A)*	13	(0.433)		54	(0.193)				
**3 (G)*	17	(0.567)		226	(0.807)				
Enzymes involved in the elimination of TAM and its metabolites									
SULT1A1									
**1/*1 (G/G)*	5	(0.333)		27	(0.193)				
**1/*2 (G/A)*	8	(0.533)		74	(0.529)		0.314	YES	0.300
**2/*2 (A/A)*	2	(0.133)		39	(0.279)				
**1 (G)*	18	(0.600)		128	(0.457)				
**2 (A)*	12	(0.400)		152	(0.543)				
UGT2B7									
**1/*1 (T/T)*	2	(0.133)		16	(0.114)				
**1/*2 (T/C)*	4	(0.267)		63	(0.450)		0.345	YES	0.370
**2/*2 (C/C)*	9	(0.600)		61	(0.436)				
**1 (T)*	8	(0.267)		95	(0.339)				
**2 (C)*	22	(0.733)		185	(0.661)				
UGT2B15									
**1/*1 (A/A)*	2	(0.133)		17	(0.121)				
**1/*2 (A/C)*	6	(0.400)		83	(0.593)		0.285	NO	0.322
**2/*2 (C/C)*	7	(0.467)		40	(0.286)				
**1 (A)*	10	(0.333)		117	(0.418)				
**2 (C)*	20	(0.667)		163	(0.582)				
Estrogen receptor. TAM therapeutic target									
***ESRA* V364E**									
364V/364V (T/T)	9	(0.600)		90	(0.643)				
364V/364E (T/A)	1	(0.067)		29	(0.207)		**0.155**	NO	**0.134**
364E/364E (A/A)	5	(0.333)		21	(0.150)				
364V (T)	19	(0.633)		209	(0.746)				
364E (A)	11	(0.367)		71	(0.254)				

**Fisher's exact test; [$]HWE (Hardy Weinberg Equilibrium); [#]Logistic regression*

***CYP3A4*1B is currently CYP3A4*1.001 according to PharmGKB (pharmgkb.org).*

Table S8: Demographic aspects of patients with vaginal bleeding (cases) and without vaginal bleeding (controls) for the retrospective study and univariate logistic regression analysis.

Variables	Cases (n=8)		Controls (n=147)		*p-value*	*p-value#*
**Anthropometric Characteristics**						
Age (years)	8	57 ± 8	145	59 ± 13	0.793**	0.610
Weight. (Kg)	8	73 ± 11	145	69 ± 15	0.309**	0.529
Height. (m)	8	1.54 ± 0.06	141	1.55 ±0.06	0.551***	0.547
BMI (Kg/m^2)	8	31 ± 3	141	29 ± 6	0.110**	0.385
**Socio-genetic gradient**						
Blood type	3		91			
AB	1		2		0.092*	**0.140**
A	1		20			
B	0		8			
O	1		61			
Number of members in the family	8	3 ± 2	146	3 ± 2	0.445**	0.547
Socioeconomic	8		146			
<$135.000.-	3		31		0.671*	0.617
$135.001-$500.000.-	4		86			
$500.001-$1.000.000.-	1		25			
>2.000.000.-	0		4			
**Risk factor's**						
Alcoholic Habit Presence	8	0	144	0	-	-
Presence of Smoking Habit	8	3	144	41	0.691*	0.593
Presence family history of some cancer	8	5	144	89	1.000*	0.969
Presence Family History of breast or ovary cancer	8	1	144	39	0.682*	0.327
**Gynecological Characteristics**						
Menarche age (years)	8	13 ± 2	138	13 ±2	0.726**	0.785
Number of Gestations	8	3 ± 1	145	3 ± 3	0.809**	0.490
Number of deliveries	8	2 ± 1	145	3 ± 2	1.000**	0.536
Number of Abortions	8	1 ± 1	145	1 ± 1	0.848**	0.665
Breastfeeding time (months)	8	3 0± 32	123	24 ± 27	0.612**	0.553
Oral Contraceptive Treatment (months)	8	31 ± 64	140	36 ± 64	0.952**	0.828
Treatment with HRT for menopause (months)	8	0	128	12 ± 48	0.216**	-
**Pathological Features**						
Age of diagnosis (years)	8	54 ± 9	146	55 ± 13	0.882***	0.881
Menopausal status						
Premenopause	4		58		0.714*	0.557
Postmenopause	4		89			
Cancer stage at diagnosis	8		155			
I	2		54		0.429*	0.516
II	4		77			
III	2		16			
Tumor Histology	8		146			
Ductal carcinoma in situ (DCis)	0		4		0.117*	-
Invasive Ductal Carcinoma (IDC)	7		127			
Invasive Lobular Carcinoma (ILC)	0		8			
Others. (IBC. IPC. etc.)	1		7			
Cell Differentiation Degree	8		131			
G1	1		39		0.690*	0.421
G2	6		69			
G3	1		23			
Treatment before to TAM	5		87			
Surgery	0		12		0.846*	0.387
Surgery + radiotherapy	2		32			
Surgery + chemotherapy	0		12			
Surgery + chemotherapy + radiotherapy	3		31			

****Student test for parametric data; ** Mann-Whitney test for non-parametric data;* * Fisher's exact test for both categorical variable; #*Logistic regression.*

Table S9.- Genotypic and allelic frequencies of *CYP2D6*4* (rs3892097), *CYP3A4*1B* (rs2740574), *CYP3A5*3* (rs776746), *SULT1A1*2* (rs9282861), *UGT2B7*2* (rs7439366), *UGT2B15*2* (rs1902023), and *ESRA* V364E (rs121913044) polymorphisms in patients with vaginal bleeding (cases) and without vaginal bleeding (controls) for a retrospective study and univariate logistic regression analysis.

**Polymorphism in genes**	Cases (n=8)			Controls(n=147)			*p-value**	[$]*HWE*	*p-value#*
Enzymes involved in the activation of TAM									
CYP2D6									
**1/*1 (G/G)*	5	(0.625)		110	(0.748)				
**1/*4 (G/A)*	3	(0.375)		33	(0.224)		0.513	YES	0.374
**4/*4 (A/A)*	0	(0.000)		4	(0.027)				
**1 (G)*	13	(0.813)		253	(0.861)				
**4 (A)*	3	(0.188)		41	(0.139)				
****CYP3A4***									
**1/*1 (A/A)*	6	(0.750)		132	(0.898)				
**1/*1B (A/G)*	2	(0.250)		14	(0.095)		0.237	YES	0.224
**1B/*1B (G/G)*	0	(0.000)		1	(0.007)				
**1 (A)*	14	(0.875)		278	(0.946)				
**1B (G)*	2	(0.125)		16	(0.054)				
CYP3A5									
**1/*1 (A/A)*	0	(0.000)		5	(0.034)				
**1/*3 (A/G)*	6	(0.750)		51	(0.347)		**0.085**	YES	**0.070**
**3/*3 (G/G)*	2	(0.250)		91	(0.619)				
**1 (A)*	6	(0.375)		61	(0.207)				
**3 (G)*	10	(0.625)		233	(0.793)				
Enzymes involved in the elimination of TAM and its metabolites									
SULT1A1									
**1/*1 (G/G)*	4	(0.500)		28	(0.190)				
**1/*2 (G/A)*	1	(0.125)		81	(0.551)		**0.025**	YES	**0.036**
**2/*2 (AA)*	3	(0.375)		38	(0.259)				
**1 (G)*	9	(0.563)		137	(0.466)				
**2 (A)*	7	(0.438)		157	(0.534)				
UGT2B7									
**1/*1 (T/T)*	0	(0.000)		18	(0.122)				
**1/*2 (T/C)*	4	(0.500)		63	(0.429)		0.775	YES	**0.154**
**2/*2 (C/C)*	4	(0.500)		66	(0.449)				
**1 (T)*	4	(0.250)		99	(0.337)				
**2 (C)*	12	(0.750)		195	(0.663)				
UGT2B15									
**1/*1 (A/A)*	1	(0.125)		18	(0.122)				
**1/*2 (A/C)*	4	(0.500)		85	(0.578)		0.876	NO	0.896
**2/*2 (C/C)*	3	(0.375)		44	(0.299)				
**1 (A)*	6	(0.375)		121	(0.412)				
**2 (C)*	10	(0.625)		173	(0.588)				
Estrogen receptor. TAM therapeutic target									
***ESRA* V364E**									
364V/364V (T/T)	3	(0.375)		96	(0.653)				
364V/364E (T/A)	1	(0.125)		29	(0.197)		**0.050**	NO	**0.081**
364E/364E (A/A)	4	(0.500)		22	(0.150)				
364V (T)	7	(0.438)		221	(0.752)				
364E (A)	9	(0.563)		73	(0.248)				

*Fisher's exact test; [$]HWE (Hardy Weinberg Equilibrium); [#]*Logistic regression*

***CYP3A4*1B is currently CYP3A4*1.001, according to PharmGKB (pharmgkb.org).*

Table S10: Multivariate logistic regression analysis or logit model for relapse, after stepwise forward and backward procedure univariate analysis.

	Coef.*	95% CI	*p-value*	*Model*
Body Mass Index (BMI)	0.158	-0.053 - 0.369	0.142	
Socioeconomic ($CLP)				
SE I: <$135.000.-	Ref.	--------	--------	
SE II: $135.001-$500.000.-	18.823	-4,802.625 - 4,840.272	0.994	
SE III: $500.001-$1.000.000.-	18.776	-4,802.674 - 4,840.226	0.994	
SE IV: >2.000.000.-	21.888	-4,799. 564 - 4,843.34	0.993	
Presence Family History of Brest Cancer (HBC)	-0.944	-3.163 - 1.276	0.405	
Number of Gestations (G)	-0.402	-1.224 - 0.420	0.338	
Number of Abortions (A)	0.526	-0.586 - 1.638	0.354	
Age of diagnosis, years (Age diag)	-0.121	-0.3118 - 0.070	0.215	
Postmenopause (Postmenop)	5.153	-0.398 - 10.704	0.069	p = 0.010
Cancer stage at diagnosis				
Stage I	Ref.	--------	--------	Pseudo R^2 = 0.495
Stage II	0.510	-2.264 - 3.284	0.718	
Stage III	5.353	0.870 - 9.836	0.019	
SULT1A1				
**1/*1 (G/G)*	Ref.	--------	--------	
**1/*2 (G/A)*	-0.272	-3.427 - 2.883	0.866	
**2/*2 (A/A)*	0.809	-2.707 - 4.325	0.652	
UGT2B15				
**1/*1 (A/A)*	Ref.	--------	--------	
**1/*2 (A/C)*	-4.585	-8.221 - 0.950	0.013	
**2/*2 (C/C)*	-1.150	-3.813 - 1.512	0.397	
Constant (β_0)	-21.870	-4,843.331 - 4,799.589	0.993	

**Logit – Cumulative standard logistic distribution (F). Coef., log(p/1-p).; 95% CI, 95% confidence interval.* Pseudo R^2, McFadden's Pseudo R squared.

Table S11: Multivariate logistic regression analysis or logit model for endometrial hyperplasia, after stepwise forward and backward procedure univariate analysis.

	**Coef.***	**95% CI**	***p-value***	***Model***
Body Mass Index (BMI)	0.147	0.010 - 0.285	0.036	p = 0.002<br><br>Pseudo R^2 = 0.42
Presence Family History of breast or ovary cancer (HBOC)	0.477	-1.736 – 2.689	0.673	
Postmenopause (Postmenop)	-1.313	-2.986 - 0.360	0.124	
Stage I	Ref.	--------	--------	
Stage II	-3.446	-5.741 - -1.150	0.003	
Stage III	-3.132	-6.136 - -0.128	0.041	
Ductal carcinoma in situ (DCis)	Ref.	--------	--------	
Invasive Ductal Carcinoma (IDC)	13.387	-3893.36 – 3920.135	0.995	
Invasive Lobular Carcinoma (ILC)	(omitted)	-	-	
Others Histology (OH)	17.433	-3889.316 – 3924.181	0.993	
CYP2D6				
**1/*1 (G/G)*	Ref.	--------	--------	
**1/*4 (G/A)*	1.512	-0.657 – 3.681	0.172	
**4/*4 (A/A)*	(omitted)	-		
****CYP3A4***				
**1/*1 (A/A)*	Ref.	--------	--------	
**1/*1B (A/G)*	-2.096	-5.706 – 1.514	0.255	
**1B/*1B (G/G)*	(omitted)	-	-	
CYP3A5				
**1/*1 (A/A)*	Ref.	--------	--------	
**1/*3 (A/G)*	1.892	-1.821 – 5.604	0.318	
**3/*3 (G/G)*	-1.037	-4.333 – 2.259	0.537	
SULT1A1				
**1/*1 (G/G)*	Ref.	--------	--------	
**1/*2 (G/A)*	-1.631	-3.728 - 0.465	0.127	
**2/*2 (A/A)*	-0.526	-2.869 – 1.818	0.660	
UGT2B15				
**1/*1 (A/A)*	Ref.	--------	--------	
**1/*2 (A/C)*	-1.071	-3.278 – 1.137	0.342	
**2/*2 (C/C)*	1.374	-0.943 – 3.690	0.245	
***ESRA* V364E**				
364V/364V (T/T)	Ref.	--------	--------	
364V/364E (T/A)	-1.701	-4.907 – 1.505	0.298	
364E/364E (A/A)	1.332	-0.516 – 3.180	0.158	
Constant (β_0)	-17.793	-3924.545 – 3888.957	0.993	

**Logit – Cumulative standard logistic distribution (F). Coef., log(p/1-p).; 95% CI, 95% confidence interval.* Pseudo R^2, McFadden's Pseudo R squared.
***CYP3A4*1B is currently CYP3A4*1.001, according to PharmGKB (pharmgkb.org).*

Table S12: Multivariate logistic regression analysis or logit model for vaginal bleeding, after stepwise forward and backward procedure univariate analysis.

	Coef.*	95% CI	*p-value*	*Model*
Body Mass Index (BMI)	0.051	-0.092 - 0.193	0.486	p = 0.014<br><br>Pseudo R^2 = 0.34
Oral Contraceptive Treatment (OCT)	-0.008	-0.026 - 0.010	0.392	
Postmenopause (Postmenop)	-0.745	-2.574 – 1.085	0.425	
CYP2D6				
**1/*1 (G/G)*	Ref.	--------	--------	
**1/*4 (G/A)*	1.876	-0.196 – 3.947	0.076	
**4/*4 (A/A)*	(omitted)	-	-	
*****CYP3A4***				
**1/*1 (A/A)*	Ref.	--------	--------	
**1/*1B (A/G)*	1.948	-0.313 – 4.209	0.091	
**1B/*1B (G/G)*	(omitted)	-		
SULT1A1				
**1/*1 (G/G)*	Ref.	--------	--------	
**1/*2 (G/A)*	-3.805	-6.594 - -1.017	0.007	
**2/*2 (A/A)*	-1.181	-3.232 - 0.869	0.259	
***ESRA* V364E**				
364V/364V (T/T)	Ref.	--------	--------	
364V/364E (T/A)	0.305	-2.262 – 2.873	0.816	
364E/364E (A/A)	2.902	0.832 – 4.973	0.006	
Constant (β_0)	-4.011	-9.253 – 1.231	0.134	

**Logit – Cumulative standard logistic distribution (F). Coef., log(p/1-p).; 95% CI, 95% confidence interval.* Pseudo R^2, McFadden's Pseudo R squared.
***CYP3A4*1B is currently CYP3A4*1.001, according to PharmGKB (pharmgkb.org).*

Table S13: Univariable logistic regression analysis for demographic and clinical characteristics of patients according to presence of ADRs (cases) and absence of ADRs (controls).

Variables	Cases (n= 116)			Controls (n=39)			*p-value*	*p-value#*
**Anthropometric Characteristics**								
Age (years)	116	58 ± 12		37	62 ± 13		0.085**	0,075
Weight. (Kg)	115	70 ± 15		38	65 ± 12		0.166**	0.084
Height. (m)	112	1.55± 0.05		37	1.55 ±0.06		0.241***	0.237
BMI (Kg/m^2)	112	29 ± 6		37	27 ± 5		0135**	0.117
**Socio-genetic gradient**								
Blood type	71			23				
AB	3			0			0.420*	0.218
A	13			8				
B	7			1				
O	48			14				
Number of members in the family	115	3 ± 2		39	3 ± 2		0.177**	0.188
Socioeconomic status	115			39				
<$135.000.-	22			12			0.118*	**0.048**
$135.001-$500.000.-	70			20				
$500.001-$1.000.000.-	19			7				
>2.000.000.-	4			0				
**Risk factor's**								
Alcoholic Habit Presence	114	0		38	0		-	-
Presence of Smoking Habit	114	37		38	7		0147*	0.088
Presence family history of some cancer	114	68		38	26		0.441*	0331
Presence Family History of breast or ovary cancer	114	32		38	8		0.524*	0.387
**Gynecological Characteristics**								
Menarche age (years)	111	13 ± 2		35	13 ± 2		0.834**	0.955
Number of Gestations	115	3 ± 2		38	4 ± 3		**0.036****	**0.029**
Number of deliveries	115	2 ± 2		38	3 ± 3		0.135**	**0.027**
Number of Abortions	115	1 ± 1		38	1 ± 1		0.391**	0.396
Breastfeeding time (months)	101	24 ± 29		30	24 ± 226		0.908**	0.934
Oral Contraceptive Treatment (months)	111	34 ± 61		37	40 ± 70		0.998**	0.610
Treatment with HRT for menopause (months)	103	9 ± 33		33	20 ± 74		0.550**	0.257
**Pathological Features**								
Age of diagnosis (years)	116	54 ± 12		38	56 ± 15		0.207***	0.205
Menopausal status	116			39				
Premenopause	50			12			0.191*	0.169
Postmenopause	66			27				
Cancer stage at diagnosis	116			39				
I	40			16			0.628*	0.676
II	63			18				
III	13			5				
Tumor Histology	116			38				
Ductal carcinoma in situ (DCis)	2			2			0.264*	0.429
Invasive Ductal Carcinoma (IDC)	101			33				
Invasive Lobular Carcinoma (ILC)	5			3				
Others. (IBC. IPC. etc.)	8			0				
Cell Differentiation Degree	106			33				
G1	31			9			0.610*	0.577
G2	55			20				
G3	20			4				
Treatment before to TAM	70			22				
Surgery	8			4			0.047*	0.224
Surgery + radiotherapy	22			12				
Surgery + chemotherapy	12			0				
Surgery + chemotherapy + radiotherapy	228			6				

****Student test for parametric data; ** Mann-Whitney test for non-parametric data;* * Fisher's exact test for both categorical variable; *#Logistic regression*

Table S14.- Genotype and allele frequencies studied polymorphisms in patients according to presence of ADRs (cases) and absence of ADRs (controls).

**Polymorphism in genes**	Cases (n= 116)			Controls (n=39)			*p-value**	[$]*HWE*	*p-value#*
Enzymes involved in the activation of TAM									
CYP2D6									
**1/*1 (G/G)*	87	(0.750)		28	(0.718)				
**1/*4 (G/A)*	25	(0.216)		11	(0.282)		0.538	YES	0.463
**4/*4 (A/A)*	4	(0.034)		0	(0.000)				
**1 (G)*	199	(0.858)		67	(0.859)				
**4 (A)*	33	(0.142)		11	(0.141)				
*****CYP3A4***									
**1/*1 (A/A)*	100	(0.862)		38	(0.974)				
**1/*1B (A/G)*	15	(0.129)		1	(0.026)		0.112	YES	**0.036**
**1B/*1B (G/G)*	1	(0.009)		0	(0.000)				
**1 (A)*	215	(0.927)		77	(0.987)				
**1B (G)*	17	(0.073)		1	(0.013)				
CYP3A5									
**1/*1 (A/A)*	3	(0.026)		2	(0.051)				
**1/*3 (A/G)*	44	(0.379)		13	(0.333)		0.656	YES	0.698
**3/*3 (G/G)*	69	(0.595)		24	(0.615)				
**1 (A)*	50	(0.216)		17	(0.218)				
**3 (G)*	182	(0.784)		61	(0.782)				
Enzymes involved in the elimination of TAM and its metabolites									
SULT1A1									
**1/*1 (G/G)*	26	(0.224)		6	(0.154)				
**1/*2 (G/A)*	60	(0.517)		22	(0.564)		0.706	YES	0.630
**2/*2 (A/A)*	30	(0.259)		11	(0.282)				
**1 (G)*	112	(0.483)		34	(0.436)				
**2 (A)*	120	(0.517)		44	(0.564)				
UGT2B7									
**1/*1 (T/T)*	13	(0.112)		5	(0.128)				
**1/*2 (T/C)*	56	(0.483)		11	(0.282)		0.079	YES	0.077
**2/*2 (C/C)*	47	(0.405)		23	(0.590)				
**1 (T)*	82	(0.353)		21	(0.269)				
**2 (C)*	150	(0.647)		57	(0.731)				
UGT2B15									
**1/*1 (A/A)*	14	(0.121)		5	(0.128)				
**1/*2 (A/C)*	68	(0.586)		21	(0.538)		0.833	NO	0.868
**2/*2 (C/C)*	34	(0.293)		13	(0.333)				
**1 (A)*	96	(0.414)		31	(0.397)				
**2 (C)*	136	(0.586)		47	(0.603)				
Estrogen receptor. TAM therapeutic target									
***ESRA* V364E**									
364V/364V (T/T)	73	(0.629)		26	(0.667)				
364V/364E (T/A)	23	(0.198)		7	(0.179)		0.929	NO	0.915
364E/364E (A/A)	20	(0.172)		6	(0.154)				
364V (T)	169	(0.728)		59	(0.756)				
364E (A)	63	(0.272)		19	(0.244)				

**Fisher's exact test; [$]HWE (Hardy Weinberg Equilibrium); [#]Logistic regression.*
***CYP3A4*1B is currently CYP3A4*1.001, according to PharmGKB (pharmgkb.org).*

References to supplementary material

Bhasker, C R, W McKinnon, A Stone, A C Lo, T Kubota, T Ishizaki, and J O Miners. 2000. "Genetic Polymorphism of UDP-Glucuronosyltransferase 2B7 (UGT2B7) at Amino Acid 268: Ethnic Diversity of Alleles and Potential Clinical Significance." *Pharmacogenetics* 10 (8): 679–85. http://www.ncbi.nlm.nih.gov/pubmed/11186130.

Cavalli, S A, M H Hirata, and R D Hirata. 2001. "Detection of MboII Polymorphism at the 5' Promoter Region of CYP3A4." *Clin Chem* 47 (2): 348–51. http://www.ncbi.nlm.nih.gov/pubmed/11159790.

Gough, A C, J S Miles, N K Spurr, J E Moss, A Gaedigk, M Eichelbaum, and C R Wolf. 1990. "Identification of the Primary Gene Defect at the Cytochrome P450 CYP2D Locus." *Nature* 347 (6295): 773–76. https://doi.org/10.1038/347773a0.

Hiratsuka, Masahiro, Yoh Takekuma, Naomi Endo, Kaori Narahara, Samar Ismail Hamdy, Yukinaga Kishikawa, Masaki Matsuura, Yasuyuki Agatsuma, Tomoko Inoue, and Michinao Mizugaki. 2002. "Allele and Genotype Frequencies of CYP2B6 and CYP3A5 in the Japanese Population." *European Journal of Clinical Pharmacology* 58 (6): 417–21. https://doi.org/10.1007/s00228-002-0499-5.

Lévesque, E, M Beaulieu, M Green, T Tephly, A Bélanger, and D Hum. 1997. "Isolation and Characterization of UGT2B15(Y85): A UDP-Glucuronosyltransferase Encoded by a Polymorphic Gene." *Pharmacogenetics* 7 (4): 317–25. http://www.ncbi.nlm.nih.gov/pubmed/9295060.

Lin, Yvonne S, Amy L S Dowling, Sean D Quigley, Federico M Farin, Jiong Zhang, Jatinder Lamba, Erin G Schuetz, and Kenneth E Thummel. 2002. "Co-Regulation of CYP3A4 and CYP3A5 and Contribution to Hepatic and Intestinal Midazolam Metabolism." *Molecular Pharmacology* 62 (1): 162–72. http://www.ncbi.nlm.nih.gov/pubmed/12065767.

McInerney, E M, B A Ince, D J Shapiro, and B S Katzenellenbogen. 1996. "A Transcriptionally Active Estrogen Receptor Mutant Is a Novel Type of Dominant Negative Inhibitor of Estrogen Action." *Molecular Endocrinology (Baltimore, Md.)* 10 (12): 1519–26. http://www.ncbi.nlm.nih.gov/pubmed/8961262.

Muñoz, S, V Vollrath, M P Vallejos, J F Miquel, C Covarrubias, A Raddatz, and J Chianale. 1998. "Genetic Polymorphisms of CYP2D6, CYP1A1 and CYP2E1 in the South-Amerindian Population of Chile." *Pharmacogenetics* 8 (4): 343–51. http://www.ncbi.nlm.nih.gov/pubmed/9731721.

NCBI. n.d. "Single Nucleotide Polymorphism, DbSNP."

Paris, P L, P A Kupelian, J M Hall, T L Williams, H Levin, E A Klein, G Casey, and J S Witte. 1999. "Association between a CYP3A4 Genetic Variant and Clinical Presentation in African-American Prostate Cancer Patients." *Cancer Epidemiology, Biomarkers & Prevention : A Publication of the American Association for Cancer Research, Cosponsored by the American Society of Preventive Oncology* 8 (10): 901–5. http://www.ncbi.nlm.nih.gov/pubmed/10548319.

Raftogianis, R B, T C Wood, D M Otterness, J A Van Loon, and R M Weinshilboum. 1997. "Phenol Sulfotransferase Pharmacogenetics in Humans: Association of Common SULT1A1 Alleles with TS PST Phenotype." *Biochemical and Biophysical Research Communications* 239 (1): 298–304. https://doi.org/10.1006/bbrc.1997.7466.

Restrepo, Juan Gonzalo, Carmen Martínez, Augusto García-Agúndez, Elmer Gaviria, José Julio Laguna, Elena García-Martín, and José A G Agúndez. 2011. "Cytochrome P450 CYP2B6 Genotypes and Haplotypes in a Colombian Population: Identification of Novel Variant CYP2B6 Alleles." *Pharmacogenetics and Genomics* 21 (12): 773–78. https://doi.org/10.1097/FPC.0b013e32834b3efc.

Roco, Angela, Luis Quiñones, José A G Agúndez, Elena García-Martín, Valentina Squicciarini, Carla Miranda, Joselyn Garay, et al. 2012. "Frequencies of 23 Functionally Significant Variant Alleles Related with Metabolism of Antineoplastic Drugs in the Chilean Population: Comparison with Caucasian and Asian Populations." *Frontiers in Genetics* 3 (January): 229. https://doi.org/10.3389/fgene.2012.00229.

Roy, Jean-Nicholas, Julie Lajoie, Lynn S Zijenah, Azemi Barama, Charles Poirier, Brian J Ward, and Michel Roger. 2005. "CYP3A5 Genetic Polymorphisms in Different Ethnic Populations." *Drug Metabolism and Disposition: The*

Biological Fate of Chemicals 33 (7): 884–87. https://doi.org/10.1124/dmd.105.003822.

Thibaudeau, Jean, Johanie Lépine, Jelena Tojcic, Yannick Duguay, Georges Pelletier, Marie Plante, Jacques Brisson, et al. 2006. “Characterization of Common UGT1A8, UGT1A9, and UGT2B7 Variants with Different Capacities to Inactivate Mutagenic 4-Hydroxylated Metabolites of Estradiol and Estrone.” *Cancer Research* 66 (1): 125–33. https://doi.org/10.1158/0008-5472.CAN-05-2857.
